# Oral Health and Drug Use Among People Experiencing Homelessness: A Community-Based Cross-Sectional Study

**DOI:** 10.7759/cureus.115990

**Published:** 2026-09-09

**Authors:** Shitanshu Malhotra, Mohd Akram Faraz, Shailja Shukla, Chaman Zehra

**Affiliations:** 1 Department of Public Health Dentistry, Career Post Graduate Institute of Dental Science and Hospital, Lucknow, IND

**Keywords:** drug use, homelessness, oral health, polysubstance use, tobacco

## Abstract

Introduction: The health vulnerability that homeless individuals encounter, in which poor nutrition, irregular hygiene behaviours, social exclusion, and limited access to preventive dental care converge with high rates of substance use, is a major factor in the acceleration of oral disease progression. Xerostomia, bruxism, poor diet, delayed care-seeking, and trauma are other ways that drug use exacerbates oral health issues, but the extent to which these interactions occur in the homeless population is not well quantified in low- and middle-income countries.

Objective: The aim of this study was to use outreach-based sampling to describe oral health conditions and drug-use patterns among people experiencing homelessness and to examine the relationship between substance use and oral health outcomes.

Materials and methods: The study involved a community-based, cross-sectional study of homeless persons living on the roads and in public places in Lucknow city. Caries experience was recorded as Decayed, Missing, and Filled Teeth (DMFT), and Plaque Index and Gingival Index scores were recorded as means of the teeth/surfaces examined. The examiner examined the oral mucosa for the presence of common oral lesions and their location. The data were entered into a spreadsheet and analysed using standard statistical software. The chi-square test or Fisher's exact test was used to test associations for categorical data and the t-test or Wilcoxon rank-sum test for continuous data.

Results: A total of 300 homeless people (PEH) were assessed, and 246 (82.0%) reported drug use (including any tobacco use), while 186 (62.0%) reported any alcohol use, 138 (46.0%) of whom reported hazardous alcohol use. Higher untreated decay was associated with increased risk for substance use. Lesions were more common among opioid users, 22/66 (33.3%), than non-users, 35/234 (15.0%). The results of this study indicate that the oral disease burden experienced by people who are homeless was high, with a high prevalence of untreated caries, tooth loss, periodontitis, and reported pain, as well as a high prevalence of substance use and tobacco use in this population. Untreated caries was strongly correlated with high-risk substance use, tobacco use with periodontitis, and hazardous alcohol use with poorer plaque control, with a clear protective gradient for more frequent toothbrushing.

## Introduction

Homelessness is an emerging public health problem that involves health and social disadvantage, poverty, and lack of access to health care [1]. An individual who is homeless (IWH) is likely to have multiple simultaneous burdens, such as mental illness, substance dependence, an unstable living situation, and malnutrition [2]. These individuals share unique vulnerabilities that make them much more likely than others to have poor oral health. Oral diseases in this group are often severe, painful, and untreated for years, as they have difficulty accessing timely and preventive dental care. This means that small cavities often worsen, leading to fractured teeth, tooth abscesses, and tooth loss [3].

There is also a strong correlation between homelessness and substance use. Xerostomia, tooth wear, and aggressive caries patterns are strongly associated with stimulants, including methamphetamine and cocaine, with a greater association with the latter [4]. Poor self-care practices, hyposalivation, and sweetened beverage consumption are correlated with opioid dependence. Tobacco smoking is a significant habit among people experiencing homelessness (PEH) and increases periodontal disease, tooth loss, and oral lesions. These conjunctive exposures contribute to the deterioration of oral tissues and establish a vicious circle of pain, self-neglect, drug dependency, and functional impairment [5]. For this population, outreach-based public health strategies delivered where PEH gather (e.g., shelters, food distribution centres, encampments, or the street) are more appropriate. A second type of sampling, outreach sampling, is also required to produce valid epidemiological information, since only a small, unrepresentative fraction of homeless people attend clinics voluntarily [6].

It is important to note that data on oral health among PEH are limited in countries in which informal settlements and transitional homelessness are common, such as India. The majority of available surveys are either general poverty surveys or clinic-attending surveys [7]. There is also a growing problem with substance use among Indian PEH, as they have easy access to alcohol, smokeless tobacco, inhalants, and low-cost psychoactive drugs, with both direct and indirect impacts on oral health. Without community-level outreach and appropriate sampling to ensure that the size and distribution of disease are not underestimated, public health planning and resource allocation may be off target [8].

Thus, a community-based, cross-sectional approach using community outreach sampling is important to provide a realistic estimate of both oral health and drug-use patterns among PEH. This study aims to address this evidence gap by describing the state of oral health and drug-use patterns among PEH using a community-based, cross-sectional approach with community outreach sampling.

Aim and objective

This study aimed to describe the nature and extent of oral health conditions and drug-use patterns among PEH and to examine the relationship between oral health and drug use among PEH through outreach-based sampling.

## Materials and methods

Study design and setting

This community-based, cross-sectional study was conducted through outreach dental camps organised by Career Post Graduate Institute of Dental Sciences & Hospital, Lucknow. The study was conducted under ethical approval number CPGIDSH/25/83, and the study period extended from September 2025 to November 2025. The study population comprised PEH who were living on the streets or in public spaces in Lucknow. Individuals present at the campsites were approached and invited to participate. The Institutional Ethics Committee of Career Post Graduate Institute of Dental Sciences & Hospital, Lucknow, approved the study protocol. Written informed consent was obtained from all participants. For participants with limited literacy, the information sheet was read aloud, and witnessed consent was obtained.

Adults aged ≥18 years who were currently experiencing homelessness and residing on the streets or in public spaces were eligible for inclusion. Individuals with a medical emergency or intoxication at the time of contact, as well as completely edentulous individuals without a mucosal complaint, were excluded.

The dental camps were conducted on pre-arranged days throughout the study period at known meeting points for homeless individuals, including areas near shelters, bus and railway stations, and marketplaces. Registration, informed consent, interview, and clinical examination were completed during a single visit at each camp. All camps were attended by the same examiner to ensure consistency in data collection and clinical assessment.

Sampling and data collection

Consecutive convenience sampling was used to recruit participants at each outreach camp. A total of 300 PEH were assessed and interviewed. A brief structured questionnaire was administered by the examiner to collect information on substance-use patterns and oral hygiene practices. Substance use was assessed using the Alcohol, Smoking and Substance Involvement Screening Test (ASSIST) [9], while hazardous alcohol consumption was evaluated using the Alcohol Use Disorders Identification Test-Consumption (AUDIT-C) [10].

A chairside oral examination was subsequently performed using a mouth mirror and Community Periodontal Index (CPI) probe under a portable light source. Dental caries experience was recorded using the Decayed, Missing and Filled Teeth (DMFT) index according to the World Health Organization Oral Health Surveys: Basic Methods [11]. Periodontal status was assessed using the CPI [12]. Oral hygiene status was evaluated using the Silness and Löe Plaque Index [13], and gingival inflammation was assessed using the Löe and Silness Gingival Index [14]. Plaque and gingival indices were recorded as the mean scores for the examined teeth/surfaces. The oral mucosa was systematically examined for common lesions, and the presence and anatomical location of lesions were recorded. Urgent oral conditions, including swelling, abscesses, and trauma, were documented, and an immediate referral note was provided when indicated.

Statistical analysis

Data were entered into a spreadsheet and analysed using IBM SPSS Statistics for Windows, Version 32 (Released 2026; IBM Corp., Armonk, New York). Continuous variables were summarised as mean ± standard deviation (SD) or median with interquartile range (IQR), as appropriate, while categorical variables were presented as frequencies and percentages. The chi-square test was used to assess associations between categorical variables, and the independent-samples t-test was used to compare continuous variables between groups, as appropriate. All statistical tests were two-sided, and a p-value <0.05 was considered statistically significant.

## Results

A total of 300 PEH were examined. The average age was 36.8 ± 11.2 years, representing a primarily young to middle-aged population. There were 234 males (78.0%) and 66 females (22.0%), with males being generally more visible in unsheltered environments. Brushing ≤1/day was reported by 216 (72.0%), while 72 (24.0%) brushed twice daily and 12 (4.0%) brushed ≥3/day. Thirty-six (12.0%) had a dental appointment in the previous year, indicating significant barriers to access and delayed care. Regarding drug-use patterns, any tobacco use was reported by 246 (82.0%) and any alcohol use by 186 (62.0%); hazardous alcohol use (AUDIT-C high) was reported by 138 (46.0%). Other substances included cannabis in 84 (28.0%), opioids in 66 (22.0%), stimulants in 54 (18.0%), inhalants in 30 (10.0%), and sedatives in 36 (12.0%). The number of cases with any high-risk use (ASSIST moderate/high) was 174 (58.0%). Smoking was the main route of administration (165, 55.0%), followed by the oral route (84, 28.0%), snorting (30, 10.0%), and injecting (21, 7.0%), with multiple harm profiles present for oral tissues.

The oral health outcomes are presented in Table 1. Untreated caries was present in 204 (68.0%) participants. The mean DMFT was 8.6 ± 5.1, with the majority comprising missing teeth (M 4.5 ± 4.2) and decayed teeth (D 3.7 ± 3.0); there were very few filled teeth (F 0.4 ± 0.9), which is typical of pain-driven, extraction-based care. The mean Plaque Index was 1.90 ± 0.50, and the mean Gingival Index was 1.60 ± 0.50, indicating a poor level of plaque control and a moderate level of gingival inflammation. Periodontitis (CPI ≥ 3) was observed in 156 (52.0%). Mucosal lesions occurred in 57 (19.0%) participants (ulcers, 18 (6.0%); candidiasis, 21 (7.0%); leukoplakia-like lesions, 12 (4.0%); and angular cheilitis, 15 (5.0%)). There was a functional impact, as indicated by 132 (44.0%) participants reporting oral pain and 117 (39.0%) reporting chewing difficulty in the past week. There was a positive association between high-risk substance use and untreated caries, as reported in Table 2. Caries was more frequent among high-risk users: 136/174 (78.2%) versus 68/126 (54.0%) in the low-risk/none group, with a significant difference (χ² = 20.9, df = 1, p < 0.001). This shows that there is a relationship between increased substance-use risk and an increased burden of untreated decay.

**Table 1:** Oral Health Outcomes Among Study Participants Untreated caries, periodontitis, oral pain, and chewing difficulty were common, while the low mean number of filled teeth indicated limited restorative care. Mucosal lesions were also observed in a substantial proportion of participants. *“Any mucosal lesion” refers to participants presenting with at least one oral mucosal lesion. Individual lesion categories are not mutually exclusive; therefore, participants may have presented with more than one type of lesion. DMFT: decayed, missing, and filled teeth; SD: standard deviation; CPI: Community Periodontal Index.

Outcome	Value
Untreated dental caries (≥1 active lesion)	204 (68.0%)
Mean DMFT ± SD	8.6 ± 5.1
Mean decayed (D) ± SD	3.7 ± 3.0
Mean missing (M) ± SD	4.5 ± 4.2
Mean filled (F) ± SD	0.4 ± 0.9
Mean Plaque Index (Silness–Löe) ± SD	1.90 ± 0.50
Mean Gingival Index (Löe–Silness) ± SD	1.60 ± 0.50
Periodontitis (CPI ≥3 in ≥1 sextant)	156 (52.0%)
Any mucosal lesion*	57 (19.0%)
Ulcerations	18 (6.0%)
Candidiasis	21 (7.0%)
Leukoplakia-like lesions	12 (4.0%)
Angular cheilitis	15 (5.0%)
Oral pain in the last 7 days	132 (44.0%)
Difficulty chewing	117 (39.0%)

**Table 2:** Association of High-Risk Drug Use and Untreated Caries OR = 3.05 (95% CI: 1.85-5.04). ASSIST: Alcohol, Smoking and Substance Involvement Screening Test.

Group	Untreated Caries, n/N (%)
High risk (ASSIST moderate/high)	136/174 (78.2%)
Low risk/none	68/126 (54.0%)
χ² (df), p	χ² = 20.9 (1), p < 0.001

As highlighted in Table 3, there was an association between hazardous alcohol use and plaque levels. The mean Plaque Index was higher among hazardous drinkers (2.02 ± 0.48) than among those with non-hazardous/no alcohol use (1.75 ± 0.49), with a significant difference (t = 5.0, df = 298, p < 0.001). The findings indicate that those who consume alcohol at hazardous levels may have poorer oral hygiene. 

**Table 3:** Association of Hazardous Alcohol Use and Plaque Index AUDIT-C: Alcohol Use Disorders Identification Test-Consumption.

Group	Mean Plaque ± SD
Hazardous alcohol (AUDIT-C high)	2.02 ± 0.48
Non-hazardous/none	1.75 ± 0.49
t (df), p	t = 5.0 (298), p < 0.001

Table 4 shows the association between opioid use and mucosal lesions. Lesions were more common among opioid users, 22/66 (33.3%), than among non-users, 35/234 (15.0%), yielding a significant association (χ² = 9.9, df = 1, p = 0.002). Potential mechanisms include xerostomia, nutritional compromise, and trauma-related ulcerations.

**Table 4:** Association of Opioid Use and Mucosal Lesion

Group	Lesion, n/N (%)
Opioid users	22/66 (33.3%)
Non-opioid users	35/234 (15.0%)
χ² (df), p	χ² = 9.9 (1), p = 0.002

## Discussion

This community-based outreach project showed a significant burden of untreated dental caries among PEH (68%), with a mean DMFT score of 8.6 ± 5.1, and only 12% of the population had a dental visit in the past year. The results of this study are in line with the oral health needs assessment undertaken by Yusuf et al. in London, which found high levels of unmet dental needs, poor oral hygiene habits, and low attendance at dental services among PEH. They also found that more than half of the participants reported brushing their teeth fewer than two times a day and that structural factors such as stigma, lack of stable housing, and competing priorities were significant factors limiting oral health care, supporting the interpretation of the present findings that homelessness creates persistent barriers to preventive oral health care [15].

The low dental service utilisation rate seen in the current study is similar to that reported by Figueiredo et al. in Toronto, where only 32% of homeless adults accessed dental services in the last year, 75% reported having untreated dental problems, and most dental care was sought for emergency treatment. While there was a similar pattern of untreated disease across countries, the considerably lower attendance rate in the present study (12%) implies that access to routine dental care may be even more constrained in low-resource settings.

The finding that missing teeth made up the largest proportion of the DMFT index, with very few restored teeth, is consistent with the findings of Daly et al. (1998), which showed that although homeless people had extensive untreated decay and periodontal disease, very few completed comprehensive dental treatment despite extensive outreach services. Emergency care alone is insufficient to address accumulated treatment needs, as highlighted by their findings, in keeping with the recommendations of the current research for continued outreach-based prevention and restorative care [16].

The relationship between high-risk substance use and untreated dental caries (78.2% vs. 54.0%; p < 0.001), along with the higher prevalence of oral mucosal lesions among opioid users, was similar to findings from the HOPE HOME Study, which found significant relationships between alcohol use, cocaine use, smoking, and severe tooth loss in older homeless adults. Findings from both studies support the relationship between substance use and homelessness and how these factors may exacerbate poor oral health through biological effects, oral self-care issues, poor nutrition, and decreased access to oral health care [17].

Finally, the present results corroborate the most recent synthesis of evidence presented in the systematic review by John et al., which found that many PEH struggle with severe and multiple disadvantage (SMD), including homelessness, substance use, smoking, and poor oral health. They highlighted that interventions combining housing support with substance-use and oral health services were more likely to have positive health outcomes than dental-only interventions. Likewise, the current study proposes that outreach distribution of oral hygiene supplies, problem-focused dental care on the day of outreach, and brief substance-use interventions could serve as a viable strategy to help reduce this syndemic among PEH [18].

Tobacco use was the most frequently reported substance-use exposure in this population, with 82.0% of participants reporting tobacco use. This finding is particularly relevant given the substantial prevalence of periodontal disease, with 52.0% of participants meeting the study definition of periodontitis. Although the present study did not formally test the association between tobacco use and periodontitis, the coexistence of these findings highlights the importance of incorporating tobacco-use assessment and cessation support into oral health outreach programmes for PEH.

Limitations

This study has a few limitations. Given its cross-sectional design, causal inferences regarding the association between substance use and oral health outcomes are not possible. Results from outreach camps in one city may not be applicable to other homeless populations because of the use of consecutive convenience sampling. Some of the information on drug use and oral hygiene practices was self-reported and may be subject to recall and social desirability bias. Furthermore, clinical examinations were conducted by one examiner without radiographic evaluation, which could have led to the underestimation of some oral conditions. The exclusion of individuals who were intoxicated at the time of contact may have introduced selection bias and could have resulted in an underestimation of substance-use prevalence, particularly among individuals with more severe or active substance use. Despite these limitations, the community-based outreach approach offers real-world evidence about the oral health burden and substance-use patterns of PEH and highlights the importance of integrated preventive and treatment strategies.

## Conclusions

In this community-based outreach study, a high burden of oral disease was observed, with oral disease indicators such as untreated caries, tooth loss, periodontitis, and oral pain, along with widespread tobacco and other substance use, among PEH. A strong association was found between high-risk substance use and untreated caries, tobacco use and periodontitis, and hazardous alcohol use and poorer plaque control; a clear protective gradient was seen with more frequent toothbrushing. The results confirm the importance of low-barrier, trauma-informed dental care, along with peer harm-reduction and addiction support, such as mobile clinics, same-day relief care and fluoride/HPV-aligned prevention, brief motivational counselling, and active referral pathways. The “structural enablers” to facilitate uptake and continuity include fee waivers, flexible timing, outreach recall, and working with shelters and peer advocates. This study uses a cross-sectional approach and partly self-reported data, as well as a single examiner, which restricts causal inferences and may lead to some under-representation of subgroups. Future longitudinal and interventional studies should be conducted to test integrated models of dental and substance-use care and to assess cost-effectiveness and quality-of-life outcomes. Overall, targeted outreach, combined with integrated oral and addiction care, can have a real impact on decreasing disease, pain, and functional impairment in this highly marginalised group.
